# Genomic and Transcriptomic Analyses of Bioluminescence Genes in the Enope Squid *Watasenia scintillans*

**DOI:** 10.1007/s10126-020-10001-8

**Published:** 2020-10-24

**Authors:** Masa-aki Yoshida, Junichi Imoto, Yuri Kawai, Satomi Funahashi, Ryuhei Minei, Yuki Akizuki, Atsushi Ogura, Kazuhiko Nakabayashi, Kei Yura, Kazuho Ikeo

**Affiliations:** 1 Marine Biological Science Section, Education and Research Center for Biological Resources, Faculty of Life and Environmental Sciences, Shimane University, Oki, Japan; 2 Department of Genomics and Evolutionary Biology, National Institute of Genetics, Mishima, Japan; 3 Present Address: Fisheries Data Sciences Division, Fisheries Resources Institute, Japan Fisheries Research and Education Agency, Fukuura 2-12-4, Kanazawa, Yokohama, Kanagawa 236-8648 Japan; 4 Graduate School of Humanities and Sciences, Ochanomizu University, Tokyo, Japan; 5 Department of Computer Bioscience, Nagahama Institute of Bio-Science and Technology, Nagahama, Japan; 6 Department of Maternal-Fetal Biology, Research Institute, National Center for Child Health and Development, Tokyo, Japan; 7 School of Advanced Science and Engineering, Waseda University, Tokyo, Japan

**Keywords:** Firefly squid, Cephalopod, RNA editing, 3D modelling, Repetitive elements

## Abstract

*Watasenia scintillans*, a sparkling enope squid, has bioluminescence organs to illuminate its body with its own luciferase activity. To clarify the molecular mechanism underlying its scintillation, we analysed high-throughput sequencing data acquired previously and obtained draft genome sequences accomplished with comparative genomic data among the cephalopods. The genome mapped by transcriptome data showed that (1) RNA editing contributed to transcriptome variation of lineage specific genes, such as *W. scintillans* luciferase, and (2) two types of luciferase enzymes were characterized with reasonable 3D models docked to a luciferin molecule. We report two different types of luciferase in one organism and possibly related to variety of colour types in the *W. scintillans* fluorescent organs.

**Electronic supplementary material:**

The online version of this article (10.1007/s10126-020-10001-8) contains supplementary material, which is available to authorized users.

## Introduction

Around the Japanese sea, especially off Toyama, the appearance of shoals of the sparkling enope squid, *Watasenia scintillans*, heralds the beginning of spring. The *W. scintillans* is also known as the “firefly squid,” which is important for both tourism and the fishing industry in Japan. *W. scintillans* emits light from three different types of photophores (Inamura et al. 1990). Five light organs are located around each eye, and a cluster of three light organs is located at the tip of the arms. The squid also has dermal light organs producing ambient, greenish light. The dermal light organs are utilized for counter-illumination camouflage to match the brightness making it difficult for predators below to detect the squid (Young and Roper 1977). Young et al. (1979) reported that *Enoploteuthis*, the closest relative of *W. scintillans*, has dermal light organs similar to the ones of *W. scintillans*. The members of the Enoploteuthidae seems to have a common mechanism of bioluminescence. Several researches in the past have established the molecular mechanism of illumination that coelenterazine disulfate is the luciferin substrate, and the reaction requires ATP, Mg^2+^ and molecular oxygen (Tsuji 2002; Tsuji 2005; Teranishi and Shimomura 2008; Goto et al. 1974; Inoue et al. 1975; Inoue et al. 1976).

In addition to the study of luminescence, cephalopod molluscs, especially squids, have fascinated many researchers studying not only in the field of fishery science but also in the field of molecular biology, especially neuroscience, visual physiology and biophysics (Schwiening 2012; Seidou et al. 1990; Hara and Hara 1980; Shichida and Matsuyama 2009; Murakami and Kouyama 2008). To fulfil the demand of the resources, the sparkling enope squid has played a decent role because collection of the squid is exceptionally stable compared with other deep-sea squids that are usually difficult to collect. However, the recent advance genome biology has not blessed this species.

Lack of genome information had prevented us from understanding the genetic basis of cephalopod biology. The report of octopus genome from Albertin et al. (2015) has finally advanced the understanding of Cephalopoda class. The genome analysis revealed three notable characteristics of the octopus genome: (1) highly rearranged genome with transposable element expansion, (2) lineage specific duplication of certain types of genes and (3) whole transcript-wide adenosine to inosine (A-to-I) RNA editing (Alon et al. 2015; Liscovitch-Brauer et al. 2017). These characteristics featured the octopus genome as quite different from metazoan genomes. However, it is not clear whether these characteristics are specific to octopuses or to cephalopods including squids and cuttlefish. Squid and cuttlefish, different major lineage of the cephalopods, show similar body plans and morphology to the octopuses (Young 1971) but diversified over 270–284 million years ago (Mya) (Kröger et al. 2011; Vinther et al. 2012). The timescale is comparable with the time of the appearance of dinosaurs or mammals (324.7 Mya, dos Reis et al. 2015). Hence, comparative genomic studies among octopus, squid and cuttlefish may accentuate cephalopod commonalities with differences to other molluscs and spotlight the specific features among them.

The average genome size of the cephalopods known to date is slightly bigger than that of other molluscan species (Gregory 2019). We have estimated the genome size of the enope squid by comparing the mean copy number of mitochondria per cell and found that the haploid genome size was about 4.78 giga base pair (Gbp) (Hayashi et al. 2016). The size is bigger than the known genome sizes of squids (Gregory 2019). Recent genome analysis has revealed that the regions with three giga base in total in the octopus genome have been the result of enormous expansion of repetitive sequences, such as transposons, contributing nearly 45% of the genome (Albertin et al. 2015). Such repetitive elements (REs) often cause genomic rearrangement across the chromosomes (Oliver and Greene 2009). For instance, the inserts of REs were estimated as a cause of disruption of macrosynteny of the genome and resulted in the loss of the HOX gene cluster in octopus (Albertin et al. 2015). The question to be addressed is how REs arose and expanded across the cephalopod genomes during the evolution. Typically, types of causal transposable elements (TEs) show the history that the species has followed and are related to the characteristics of the organism. For example, Alu-type short interspersed nuclear element (SINE), which is the most abundant repeat sequence found in human genome, occupies more than 10% of the genome sequence (Lander et al. 2001). Despite the abundance, a major burst of the Alu amplification was estimated to have happened 25–50 Mya based on the human Alu subfamily sequence diversity (Shen et al. 1991). TEs are the major part of REs in the cephalopod genomes (da Fonseca et al. 2020) and undergo a rapid increase in copy number in some animal clades, resulting in the increase of the genome size through TE amplification and, in some cases, in causing loci rearrangements through TE insertions. The former was already observed in squid and in octopus (Yoshida et al. 2011; Albertin et al. 2015), and the latter was suggested to have happened as large-scale genomic rearrangements in the genome of California two-spot octopus *Octopus bimaculoides* (Albertin et al. 2015). It is considered that the timing of its appearance is greatly related to the reorganization of the genome; however, burst timing of REs are uncertain in the cephalopods even before/after squid-octopod divergence.

Another characteristic of cephalopod genetic system is the existence of extensive RNA editing (Liscovitch-Brauer et al. 2017). The events of RNA editing itself have been discovered in wide range of organisms including humans, *Drosophila* and so forth. However, numerous RNA-editing sites were conserved between squids and octopuses, especially in transcripts of nervous system, which is quite contrary to the situation in mammals and *Drosophila*. In humans, for instance, the locations of RNA editing in only 25 genes are conserved across mammals (Pinto et al. 2014). In *Drosophila,* only about 65 editing sites are conserved across the *Drosophila* lineage (Yu et al. 2016). RNA editing is expected to be utilized for enhancing transcriptome variation (Liscovitch-Brauer et al. 2017).

Recently, the genome of Hawaiian bobtail squid *Euprymna scolopes*, a model cephalopod with symbiotic luminescence, has been sequenced, and the highly repetitive nature of the genome was uncovered (Belcaid et al. 2019). To solidify the characteristics of cephalopod genomes and to uncover the commonality of RNA editing in this group of organisms, we obtained the draft genome sequences of the Japanese enope squid, *W. scintillans*, which has several light organs different from the ones of *E. scolopes.* We further used a genomic approach in combination with transcriptomic approach, namely assembly of both DNA and RNA sequences, and performed comparative genomics among the cephalopods. Based on these comparative genome analyses, two characteristics of cephalopod genomes have been confirmed: (1) RE variation in *W. scintillans* is different from that of octopus, and (2) RNA editing was contributed to transcriptome variation of lineage specific genes, such as *W. scintillans* luciferase. Subsequent detailed analysis of bioluminescence showed that (3) there are at least two types of luciferases in the genome of *W. scintillans*.

## Materials and Methods

### Sample Collection and Isolation of Genomic DNA and Total RNA

*W. scintillans* caught in Toyama Bay were obtained from Hotaruika Aquarium (Namerikawa, Toyama, Japan). Genomic DNA was extracted from the brain of a single female individual (L3) by a conventional phenol/chloroform extraction protocol. Briefly, the brain tissue was lysed in 600 μl of the lysis buffer (0.1 M Tris-HCl pH 8.0, 0.2 M NaCl, 5 mM EDTA, 0.2% SDS, 0.2 mg/ml Proteinase K) at 55 °C overnight. The lysate was extracted with an equal volume of phenol/chloroform/isoamyl alcohol (25:24:1) once, and with an equal volume of chloroform once. The genome DNA was precipitated by adding an equal amount of isopropanol to the lysate, washed with 70% ethanol, dried briefly and dissolved in 1× TE. Total RNA samples were also extracted from the brain and the arm tips of the same individual using the Mixer Mill MM301 (Retsch, Düsseldorf, German) for homogenization and the E.Z.N.A. Mollusc RNA Kit (Omega Bio-tek, Georgia, United states).

### Library Preparation and Sequencing

A short-insert and two mate-pair libraries were prepared from the brain genomic DNA of an individual using TruSeq DNA sample preparation kit v2 (Illumina, San Diego, USA) and Nextera mate-pair library preparation kit (Illumina), respectively. Mate-pair libraries were prepared from two size ranges of tagmented DNA fragments, 4 kb and 8 kb in average. RNA-seq libraries were prepared from the total RNA samples of the same individual (brain and arm tips) using TotalScript RNA-Seq Kit (Epicentre, Wisconsin, USA). The DNA- and RNA-seq libraries were sequenced on HiSeq1500/2500 systems (Illumina). Paired-end read (150 bp × 2) sequencing was performed using the HiSeq Rapid SBS Kit HS. The conversion of bcl files to fastq files was performed using the CASAVA v1.8.2 (configureBclToFastq.pl) software (Illumina).

### Genome and Transcriptome Assembly

After multistage base trimming, 159.3 million read pairs from the short-insert (350 bp) library and 22.7 million and 23.4 million read pairs from two mate-pair libraries (4 kb and 8 kb) were subjected to 17-mer counting of the Illumina reads using Jellyfish v.2.2.10 (Marçais and Kingsford 2011). The k-mer histogram was processed using GenomeScope v1.0 (Vurture et al. 2017) to estimate genome size, heterozygosity and repeat content. All the same reads were also applied to *de novo* genome assembly using ALLPATHS-LG v.44837 (Gnerre et al. 2011) to generate genome contigs. Transcriptome assembly was performed by the CLC Assembly Cell (QIAGEN Bioinformatics, Hilden, Germany) independently to the draft genome. The single copy genes were assessed by BUSCO v1.1b1 with lineage option, metazoa (Simão et al. 2015). The assembled partial genome sequences were deposited in the DNA DataBank of Japan (DDBJ) under the accession numbers BLWP01000001-BLWP01491107. Raw Illumina reads for genome and transcriptome are available in the DDBJ Sequence Read Archive (DRA) under BioProject accession number PRJDB8630 and DRA accession number DRA009937, respectively. Augustus v3.2 was employed to generate an *ab initio* gene models (16,509 genes) with species option, human (Stanke et al. 2008). The protein gene model file is available as a supplementary file.

### Repeat Estimation

To detect RE sequences from the molluscan whole genome shotgun sequences, we utilized the RepeatMasker programme (http://www.repeatmasker.org, RepBase Update 13.04; Kohany et al. 2006). To identify de novo REs from the molluscan genomes, the quality-controlled sequences were repeat-masked using the RepeatMasker with species-option set as “eukaryotes” due to the absence of a molluscan repeat database. For the RE database, we utilized owl limpet *Lottia gigantea* (Simakov et al. 2013), *O. bimaculoides* (Albertin et al. 2015) and Japanese pygmy squid *Idiosepius paradoxus* (Yoshida et al. 2011), respectively. As a comparison, the proportions of the three repeat sequences of three cephalopod genomes, *O. bimaculoides*, East Asian common octopus *O. sinensis* (https://www.ncbi.nlm.nih.gov/genome/84214?genome_assembly_id=678426) and giant squid *Architeuthis dux* (da Fonseca et al. 2020), were analysed using the same procedures.

### RNA Editing Analysis

We mapped RNA-seq reads to the *W. scintillans* genome by TopHat (v2.1.0, Trapnell et al. 2009), and identified SNPs by SAMtools (v0.1.19, Li et al. 2009). We also identified polymorphic positions from the SNPs predicted above by GATK Haplotype Caller (v3.5, DePristo et al. 2011) with the default setting of the discovery mode. We implemented kallisto to estimate expression levels of RNA editing enzymes ADAR genes of each sample (Bray et al. 2016). We then extracted RNA editing site candidates where the sites show polymorphic but not SNPs by BEDTools (v.2.17.0, Quinlan and Hall 2010). We finally predicted RNA editing sites by REDItools (v1.0.4, Picardi and Pesole 2013), the curation of RNA editing sites investigation software. Numbers of RNA editing sites shared between brain and arm samples are estimated with a custom script and shown in a Venn diagram (Figure S10).

### Orthologous Analysis

We utilized *W. scintillans* gene models estimated above for the orthologous relationship analysis. We also obtained gene models of *O. bimaculoides* and *L. gigantea* from the public databases. To estimate orthologous groups, we utilized OrthoFinder (v1.0.6, Emms and Kelly 2015) that can produce orthologous group of genes in which lineage specific duplicated genes would be included in the group.

### Search for Bioluminescent Proteins

We utilized homology search for finding bioluminescent genes. We first conducted extensive search of scientific literature, internet description and databases for known bioluminescent proteins and ligands. The following are the database accession numbers either from UniProt or GenBank of the ten non-homologous representatives from bioluminescent protein groups with their origin: hetero dimer of P23146 and P19840 from a gammaproteobacterium *Photorhabdus luminescens*, P08659 from common eastern firefly *Photinus pyralis*, C6KYS2 from purpleback flying squid *Sthenoteuthis oualaniensis*, AAA29804 from sea pansy *Renilla reniformis*, CAA49754 from gregarious jellyfish *Clytia gregaria*, BAG48250 from a copepod *Metridia pacifica*, O77206 from a photosynthetic dinoflagellate *Lingulodinium polyedrum*, P17554 from sea-firefly *Vargula hilgendorfii*, Q9GV45 from a luminous shrimp *Oplophorus gracilirostris* and CAA10293 from common piddock *Pholas dactylus*. We performed a homology search of the whole amino acid sequences derived from the genome and transcriptome data of *W. scintillans* for these eleven amino acid sequences using BLAST with a cut-off of E-value < 10^−5^ and the score > 90.

### Phylogenetic Analysis of Luciferases

To figure out *W. scintillans* luciferases within the animal luciferases, wsluc1-4 and symplectin-like gene from *Watasenia*, as well as adding all sequences from one of the fireflies (firefly beetles (taxid:7049)), all drosophila sequences, all human sequences, all octopus sequences (Octopodiformes (taxid:215451)), all squid sequences (Decapodiformes (taxid:215450)), all *Lottia* sequences and transcripts from *Branchiostoma*, *Crassostrea*, *Mytilus* and *Nematostella* invertebrate adenylating enzymes were found by the NCBI web BLASTP search (as of 28 August 2020) using the *W. scintillans* luciferases as a query (e-value < 1.0 × 10^−30^). To perform multiple alignment of protein sequences, we utilized MUSCLE v3.8.1551 (Edger 2004) followed by removing suspicious residues using trimAl_v1.4beta (automated1 option; Capella-Gutiérrez et al. 2009). RaxML-NG-mpi (RAxML-NG v. 0.9.0 released on 20.05.2019, Kozlov et al. 2019) was implemented to estimate the maximum likelihood tree with all option and the best fit model tested with modeltest-ng v. 0.1.3 (WAG+G4, Darriba et al. 2020). The tree was visualized with FigTree v1.4.2 (Rambaut 2009).

### Protein Three-Dimensional Structure Modelling

A set of candidate proteins for bioluminescence identified by homology search went through homology modelling to test the structural compatibility for the luminescence function. The template protein structure of each group for the homology modelling was selected from Protein Databank (PDB) (Kinjo et al. 2018) by searching the homologue of the representative protein. Then each candidate protein sequence was aligned with the amino acid sequence of template protein using ALAdeGAP (Hijikata et al. 2011), the alignment tool specifically developed for homology modelling. Based on the alignment and the three-dimensional (3D) structure of the template protein, ten 3D structures of the candidate protein were build using MODELLER (Šali and Blundell 1993). Based on DOPE energy in MODELLER, the best structure out of the ten was selected and the reasonability of the selected 3D structure was tested on ProSA-web (Wiederstein and Sippl 2007), a tool to check the compatibility of the structure against the whole 3D structures in PDB. When the template 3D structure had luciferin or its analogue, then the molecule was docked to the modelled structure by superposing the template and target structures and was transferred the coordinates of the molecule from the template to the target structures. The reasonability of the location of the luciferin was examined by the existence of atomic clash between the protein and luciferin. When only few clashes exist between the protein and luciferin, then the structure was interpreted as reasonable.

## Results

### Genome Assembly Statistics

Total read obtained in this study (117G base pairs) gives more than 24× coverages compared with the estimated haploid genome size, 4.78 Gbp (Table S1-S2, Hayashi et al. 2016). However, de novo genome assembly unexpectedly resulted in partial genome sequences with only 649 Mbp. But we conjectured that the data is still valuable for performing a large-scale comparison of genome components among molluscs (Table S3). To confirm the genome size estimation, we performed k-mer index analysis with Jellyfish and GenomeScope. Genome unique length size estimates from the GenomeScope were 666 to 672 Mbp, in reasonable agreement with those obtained by Allpaths-LG assembly (Figure S1). The analysis using GenomeScope also indicated high heterozygosity (4.9–5.9%; Figure S1), which is consistent with the difficulty of assembly by short read. Genome haploid length estimates were 2.32 to 2.34 Gbp. This is equivalent to 18.7 in terms of k-mer coverage, which contradicts the earlier coverage estimate. We further searched for the signatures of singleton genes, which should exist in the sequence and found HOX coding sequences in our data. Four scaffolds showed significant similarity to amino acid sequences of known HOX gene cluster members (Antenapedia on scaffold_238533, Hox3 on scaffold_2002, Hox5/Sex comb reduced on scaffold_88306 and Posterior2 on scaffold_309777). On each scaffold, 11, 12, 22 and 11 raw genome reads were mapped, respectively (Table S4; Figure S2). On average, the genome data showed 11.5 times coverage. This value was also supported by the distributions of 576 singleton genes based on the BUSCO metazoa, which has a peak around 12–14 times coverage (Figure S3). Since this value is almost one and a half times by the estimates of the k-mer index, the k-mer estimates are inferred to include both heterozygous and homozygous regions. The discrepancy in the number of coverage (either 24 or 11.5) likely derived from high heterogeneity among chromosomes and among the non-coding repetitive regions, which is shown below. High heterogeneity is also expected from fewer N50 (Table S3). The assembly is still highly fragmented, but the coding regions was apparently well covered and hence can be used for protein-coding gene analyses including estimation of the amount of RNA editing sites.

### Repeat Variations

**Fig. 1:**
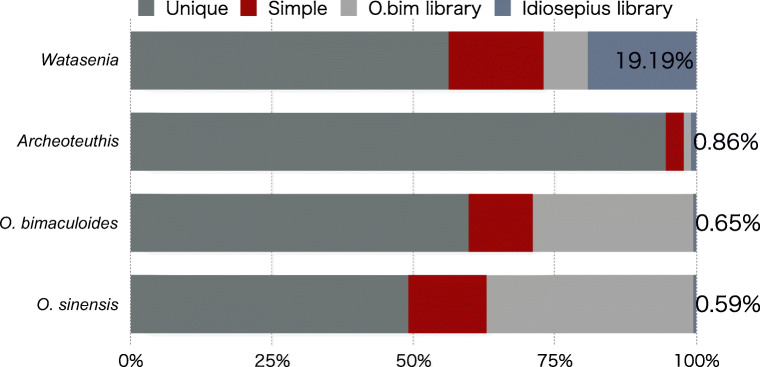
RE frequency comparison of three cephalopod genomes

To detect RE sequences and their frequency in the genome, we applied RepeatMasker, a homology-based method, on the assembly. Repeat detections were performed in the following three steps. (1) We detected simple repeats and repeat common to metazoan in the *W. scintillans* assembly by default setting (for human) and masked. Then, (2) the repeat libraries of octopus at OIST Octopus Genome site (https://groups.oist.jp/ja/molgenu/octopus-genome) were used as templates, and (3) the repeat libraries of *I. paradoxus* repeat database which we published previously (Yoshida et al. 2011) were used. By applying method 1, we estimated that the total repeat content accounted for 16.69% (10.8 Mb) of the assembly (Table S5), dominated by simple repeats. By further applying method 2, 5,149,360 bp in *W. scintillans* genome (approximately 0.79%) were estimated to comprise the repetitive sequence common to Coleoidea. By finally applying method 3, 12,458,350 bp sequences (19.2%) on *W. scintillans* scaffolds were still found to have high similarity to the *I. paradoxus* REs. The abundant number of REs after the third screening step indicates that the relative volume of REs common to squids outnumbered REs in octopus (Fig. 1; Table S5). On the other hand, the REs found in the genomes of the two octopus species are heavily biased towards the octopus common repeat library and have small contribution of *Idiosepius* library. Although *Architeuthis* is a member of oegopsid squids and the most closely related group to *W. scintillans* among the animals whose genomes are now available, the genome has a distinct pattern of REs, possibly reflecting different RE distribution or maybe the assembly procedure (Fig. 1). The amount of repeat contents given here are likely underestimated due to the incompleteness of both the assembly of the genome sequence and the contents of *I. paradoxus* repeat database.

### Orthologous Gene Analysis

To illuminate orthologous gene distribution among cephalopods, we first estimated orthologous groups of genes using *W. scintillans*, *O. bimaculoides* and *L. gigantea* as an outgroup species. As a result, we found 9535, 11,300 and 9817 orthologous gene groups in *W. scintillans*, *O. bimaculoides* and *L. gigantea*, respectively (Figure S4). Singlet genes that were found only in a specific species were not used in the following analyses. In the investigation of orthologous gene groups among the three species, we found that about 70% of the genes are conserved in molluscs. There are several percent of genes shared between *O. bimaculoides* and *L. gigantea*, but not in *W. scintillans* (2284 common genes between *O. bimaculoides* and *L. gigantea* compared with 493 ones between *W. scintillans* and *L. gigantea*; Figure S4)*.* We rather speculated that this was caused by the incompleteness in the *W. scintillans*. Therefore, we continue the following analyses from the standpoint of *W. scintillans* assembly and will not touch upon the issue of missing genes in *W. scintillans* genome. From this viewpoint, we can conclude that more than 90% of the gene groups (7003) are shared in cephalopods. Of these gene groups, we examined the sequence diversity of 1:1:1 core orthologous genes from the divergence point of *W. scintillans* and *O. bimaculoides* by setting *L. gigantea* as an ancestor. The distribution of sequence diversity deduced from the length of branches on each phylogenetic tree seems similar, but sequence diversity of the *O. bimaculoides* genes is slightly wider than those of the *W. scintillans* (Figure S5). We also conducted functional enrichment analysis of squid-specific duplicated genes in the 7003 common orthologous groups for molluscs and found that signalling pathway genes including G protein coupled receptors have been enriched in enope squid, probably reflecting their characteristics.

### Bioluminescence Genes

We searched for luciferase candidates from the *W. scintillans* genome assembly, brain RNA transcriptome and arm tip RNA transcriptome. We obtained two candidate partial sequences from brain transcriptome and four candidate partial sequences from the assembly and arm tip transcriptome. The former two sequences were very similar to the proteins reported as *W. scintillans* luciferase by Gimenez et al. (2016). Gimenez et al. reported four sequences (wsluc1, 2, 3, 4) as candidates of *W. scintillans* luciferase or enope squid luciferase derived from the RNA transcriptome of light organ in the arm tip. One of the sequences has already been registered in GenBank (Accession number: LC177398). The sequence was similar to *Photinus pyralis* (firefly) luciferase. The two sequences we found in the brain RNA transcriptome were also similar to *P. pyralis* luciferase, and hence our result strengthened the possibility that the protein is indeed the luciferase of *W. scintillans* (Figures S6, S7). To figure out *W. scintillans* luciferases within the animal luciferases, we perform phylogenetic analysis with insect luciferase genes obtained from Fallon et al. (2018) (Figure S8). The *W. scintillans* luciferases made a clade sister to firefly luciferases characterized by Fallon et al. (2018).

**Fig. 2:**
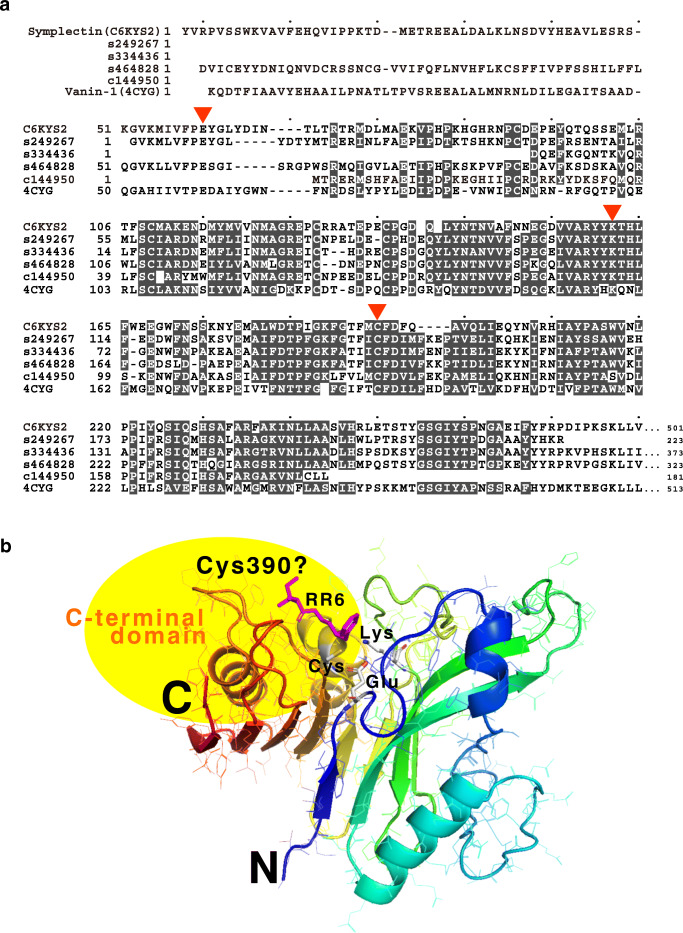
Partial sequences of symplectin-like proteins in *Watasenia scintillans* assembly (contig# s249267, s334436, s464828) and in arm tip transcriptome (c144950). **a** Amino acid sequence alignment among *Sthenoteuthis oualaniensis* symplectin (PDB ID; C6KYS2), *W. scintillans* sequences and human vanin-1 (Uniprot Accession; 4CYG). Red triangle is the location of a catalytic site on vanin-1. The number at the last of the alignment is the length of the sequence. **b** Predicted three-dimensional structure of s249267 based on the structure of human vanin-1. The sequence runs from blue to red. Three catalytic residues are shown in stick model. The structure of human vanin-1 was determined with RR6 inhibitor, which is shown in purple stick model. The catalytic residues are well aligned to RR6, which is expected to be replaced by coelenterazine in symplectin-like protein. s249267 does not have about 50 residues at the N-terminal side and 240 residues at the C-terminal side compared with human vanin-1. The C-terminal side can be located in the yellow background region, and the residue corresponding to Cys390 of *S. oualaniensis* symplectin can be located at the far side of the putative coelenterazine-binding site

The latter four candidates derived from genome assembly and arm tip RNA transcriptome had very similar sequences to *S. oualaniensis* symplectin. *S. oualaniensis* has been known to have another type of squid autofluorescence, which gives off shiny blue light (Tsuji and Leisman 1981; Chou et al. 2014). Fujii et al. (2002) singled out the agent of *S. oualaniensis* light and found symplectin as a new luciferase. Symplectin is similar to mammalian enzymes named biotinidase that hydrolyses biocytin to biotin and lysine (Cole et al. 1994). The sequence alignment between symplectin and the four candidates is shown in Fig. 2a. The enzymatic detail of symplectin is still unknown, but its human homologue vanin-1 is well studied. The 3D structure of vanin-1 has been determined with pantetheine analogue inhibitor, RR6 (Boersma et al. 2014). The putative catalytic sites of the enzyme reside on the amino acid residues pointed by red triangle in Fig. 2a. These residues correspond to Glu60, Lys163 and Cys196 in symplectin. In addition to these three residues, Cys390 is specifically important for symplectin where coelenterazine forms covalent bond (Isobe et al. 2008). Four sequences we found in this study were all partial sequences compared with the full-length symplectin, and none of the four sequences had the C-terminal domain of symplectin. Two of the four sequences lack the first catalytic residue Glu60. We speculate that these four sequences are partial sequences identified either in the incomplete genome assembly or RNA transcriptome because the identified sequences were highly similar to the N-terminal domain of symplectin and the model 3D structures of the proteins formed reasonable structure (*Z* = − 5.99 in ProSA) with RR6, which is expected to be coelenterazine on symplectin. Figure 2b shows the model of a *W. scintillans* contig (#s249267). The C-terminal domain with Cys390 should exist at the region depicted by yellow oval background in Fig. 2b, where part of the ligand protruded. Francis et al. (2017) has also found a single sequence similar to symplectin in the transcriptome data of *W. scintillans* obtained by Gimenez et al. (2016) and built a 3D structure. As in the first case in this study, the four sequences we found were also similar to *S. oualaniensis* symplectin, and hence our result strengthened the possibility of the existence of symplectin-like protein in *W. scintillans.*

### Extensive RNA Editing Events in the Luciferase

RNA editing is a post-transcriptional mechanism that involves a substitution of a specific nucleotide at the RNA level, and substitution of A-to-I occurs preferably during the post-transcriptional stage (Bass and Weintraub 1988). The combination of genome sequencing and RNA-sequencing utilizing next generation sequencing technologies made us possible to investigate RNA editing sites with ease. In cephalopods, RNA editing is known to contribute to the temperature-sensitive responding mechanism in octopus (Garrett and Rosenthal 2012). Adenosine deaminases, ADAR1 and ADAR2, are known to proceed RNA editing and are conserved in varieties of bilateral animals. Therefore, we first checked gene expression activity of ADAR1 and ADAR2 in our transcriptome data of the brain and arm and found extensive expressions of those genes in the brain rather than arm, suggesting that RNA editing events do occur in *W. scintillans* and that the events are likely more active in the brain (Table 1). In the prediction of RNA editing sites, we mapped RNA-seq reads to the genome and found that more than ten thousand of sites experienced RNA editing. As we sequenced RNA with non-stranded library construction, T-to-C substitution as well as A-to-G substitution is dominant in the RNA editing site candidates (Figure S9). To investigate the tissue specificity of the RNA editing sites, we classified A-to-G RNA editing sites by Venn diagram and found that there is little coverage of RNA editing sites and the number of sites is greater in the brain (Figure S10).

**Table 1:** Expression intensities of RNA editing enzymes (ADARs)

**Brain**	**tblastn**			**kallisto**			
		**evalue**	**bitscore**	**length**	**eff_length**	**est_counts**	**TPM**
ADAR1	TRINITY_DN1369507_c1_g9_i1	8E-105	350	2344	2135.93	2137.23	6.32754
ADAR2	TRINITY_DN1372101_c0_g5_i3	2E-173	513	1486	1277.93	211.139	1.0448
**Arm**	**tblastn**			**kallisto**			
		**evalue**	**bitscore**	**length**	**eff_length**	**est_counts**	**TPM**
ADAR1	TRINITY_DN2234624_c0_g1_i1	4E-26	110	350	163.974	8	0.332477
ADAR2	TRINITY_DN2234624_c0_g1_i1	9E-46	163	350	163.974	8	0.332477

The analyses showed that RNA editing was taken place extensively in *W. scintillans*. The finding of extensive RNA editing, especially on a novel species-specific genes of *W. scintillans*, here makes a new question arise, namely the extent of the rapidity in RNA editing evolution. To address this question, we scrutinized RNA editing in *W. scintillans* specific genes, the luciferases, characterized in this study. The luciferases are apparently forming a family with *O. bimaculoides* acyl CoA synthase. In the arm RNA transcriptome, 197 reads, giving approximately 21× coverage per site, were mapped onto the wsluc1 fragment on the *W. scintillans* genome (BLASTN, > 95% identity). We found 13 reproducible mismatches between genome and RNA from the same individual. Out of 13, three were A-to-G mismatches and 10 were cytosine to thymine mismatches. However, some RNA reads still supported A as in the DNA in those thirteen mismatch sites; therefore, those mismatches were likely the outcome of RNA editing but not complete edit sites. Those sites were close to each other and located within 30 bp. We found that two out of the three caused synonymous substitutions. An intriguing note should be taken here that wsluc1 was also expressed in the brain, but no RNA editing was found on the brain-expressed wsluc1 RNA molecule.

## Discussion

### Amplification of the Repeat Element in Cephalopod Genomes

In general, genome rearrangement across TEs was suggested to be taken place only after TE expansion. For example, analysis of primate synteny breakpoint highlighted the role of TE in partial duplication and ultimately resulted in chromosome rearrangement (Capozzi et al. 2012). In case of octopus, Albertin et al. (2015) reported that genes that are linked in other bilaterians but not in octopus are enriched in neighbouring SINE content. The SINE insertions around these genes dated to the time of tandem C_2_H_2_ expansion, which contributed to the evolution of cephalopod neural complexity and morphological innovations. If this scenario is true, common traces of the RE burst should be found throughout the genome across all cephalopods. In the present analysis, we found distinct patterns of RE types in squid and octopus genome sequences. Ceph-SINEs, which are a type of tRNA-derived SINEs originally isolated from squids (Akasaki et al. 2010), are found in common between squids and octopuses, but the frequency and patterns of those are different. Furthermore, Albertin et al. (2015) estimated that the burst of the REs was triggered between 25 and 56 Mya, which is far closer to the present compared with the timing of octopus-squid separation (∼ 270 Mya), and hence not in accordance with the idea that all cephalopod experience common SINE-based genome rearrangements. The chromosome-based macrosynteny analysis using bivalve (scallop) genome revealed correspondence between the 19 scallop chromosomes, and 17 of them presumed ancestral bilaterian linkage groups (Wang et al. 2017). Therefore, numerous genome rearrangement found in octopus should have happened after the divergence between bivalves and octopus. Hence, it remains unclear when and how genomic rearrangement occurred in the cephalopod lineage. To narrow down the time range, chromosome-level comparative genomics based on long-read sequencers should be carried out.

### Candidates for Bioluminescence in *W. scintillans*

*W. scintillans* has ability to illuminate its body without the involvement of other organisms. Tsuji demonstrated that *W. scintillans* bioluminescence is produced by a luciferin-luciferase reaction (Tsuji 1985, 2002). The responsible holoenzyme has not been characterized, but candidate proteins were found quite recently (Gimenez et al. 2016; Francis et al. 2017). In our independent study, we found two types of candidate genes in *W. scintillans* genome assembly sequence and RNA transcriptome, namely one similar to *P. pyralis* luciferase and the other similar to *S. oualaniensis* symplectin. As far as we know, there is no bioluminescent organism with two types of photoprotein.

Inamura et al. (1990) observed and Gimenez et al. (2016) pointed out that the luminescent colour of the arm emitter was slightly different from that of the body surface emitter. The arms emit bluish light, whereas the body surface emits slightly greenish light. Tsuji (2002) measured the spectral distribution of the light emitted from *W. scintillans* fourth arms and reported that it ranged from about 400 to 580 nm with a peak at 470 nm (blue). He also mentioned in the same paper that there had been a report by other workers that a small scattering of dermal organs on the ventral side had emitted a greenish-yellow light instead of blue light. It has been considered that the same molecular mechanism in different organs is used for the light emission in *W. scintillans* as suggested by Teranishi and Shimomura (2008).

*P. pyralis* luciferase emits greenish light. *S. oualaniensis* luciferase symplectin with coelenterazine was measured to emit light with wide spectral range (Chou et al. 2014), but Tsuji and Leisman (1981) measured the light spectrum of *S. oualaniensis* as a whole and found blue light emission around the wavelength of 455 nm. These pieces of evidence made us speculate that the two different types of luciferase are employed to emit different wavelength light. One may suspect the existence of fluorescent protein in *W. scintillans* that can change the wavelength of the light derived from a single luciferase. We searched for the secondary bioluminescent proteins, such as green fluorescent proteins (GFPs), using animal GFPs as queries and found no apparent hit. Different types of substrate or slight difference in amino acid residues in the binding site of substrate is known to change the wavelength, and hence two different types of luciferase may not be needed for different colour in light.

As discussed by Francis et al. (2017), comparative genomics with *W. scintillans* can open a way to discuss evolution of luminescence genes. Luciferase enzymes have extremely varied structures, mechanisms and substrate specificities across extant organisms, and hence bioluminescence should have evolved independently at least 30 times across extant organisms (Hastings 1983). This study added new candidate sequences to the diversity of bioluminescence genes.

### Extensive RNA Editing in *W. scintillans* Transcripts

Massive RNA editing was found in octopuses and squids tested so far, but not seen in the distant relative, such as the chambered nautilus and the sea hare (Liscovitch-Brauer et al. 2017). This is consistent with our analysis that shows *W. scintillans* transcriptome had extensive putative edit sites. The responsible genes, ADARs, were expressed in the same tissue, and it is reasonable to expect massive RNA editing with non-gene-specific manner. We expected that ecologically important enzymes such as the luciferases are necessary to finely tuned to the original functional complex and then avoided from the RNA editing. For example, proteins such as opsins are less variable to become adapted under certain environmental circumstances. Functionality of opsins appears to be maintained by purifying selection (Belcaid et al. 2019). Random variations by the frequent RNA editing may cause a malfunction despite purifying selection. However, RNA editing was also found in the *W. scintillans* wsluc1. The finding may support that the *W. scintillans* wsluc1 has still multifunction. The RNA editing is possibly fine-tuned in the cephalopods, characterizing that those genes may lead to find out DNA elements which positively/negatively control RNA editing machinery.

## Electronic Supplementary Material

ESM 1 (PDF 700 kb)

ESM 2 (PEP 2580 kb)
