# Supplementary material for: Genomic and Transcriptomic Analyses of Bioluminescence Genes in the Enope Squid Watasenia scintillans
Source: Mar Biotechnol (NY). 2020 Oct 24;22(6):760–71. doi: 10.1007/s10126-020-10001-8 (PMC7708342; doi:10.1007/s10126-020-10001-8)
Supplement: Supplementary file 1 — (PDF 700 kb) [file 10126_2020_10001_MOESM1_ESM.pdf]

Table S1 Raw data statistics for genome

	Total reads	Total bp
brain_TruSeqDNA_0101A.QC	150,513,222	45,153,966,600
brain_TruSeqDNA_19_0153.QC	83,708,487	25,112,546,100
brain_TruSeqDNA_2_0153.QC	68,177,737	20,453,321,100
brain_TruSeqDNA_7_0153.QC	87,899,759	26,369,927,700
Sum	390,299,205	117,089,761,500

Table S2 Raw data statistics for transcriptome

	Total reads	Total bp
brain_TotalScript_0102B	67,193,182	10,078,977,300
hand_TotalScript_0102B	57,143,544	8,571,531,600

Table S3 Partial genome assembly statistics

	#contig	The longest contig length	Total bp	Average	N50
scaffold	491,107	8,360	649,175,396	1,321.9	1,283
scaffold >1,000bp	396,231	8,360	558,932,387	1,410.6	1,365
scaffold >2,000bp	37,004	8,360	93,540,807	2,527.9	2,432

Table S4 Unique gene coverage on the Hox gene regions

**Coding regions**	**scaffold_238533.m.16.Antpout GTCCCCACCGGAAAAGAGGCCGA**	**scf12022_Hox3reg2 ACGATTTTGAAACCAGATTTTGA**	**scaffold_88366.m.61.Hox5/Scr.out TGAGTTCGTATTTCGTAAATTCG**	**scaffold_309777.m.31.Post2.out TGGAACCGACTAATAACCTGTTA**	**mean**
Frequency	11	12	22	11	11.5

	Watasenia		*O. bimaculoides*		*Lottia*	
	count	per1K	count	per1K	count	per1K
Retro	12,676	0.20	1,810,128	0.76	47,426	0.13
SINEs	8128	0.13	1146791	0.48	26,956	0.06
LINE	3723	0.06	657389	0.28	20,470	0.06
LTR/ERVs	825	0.01	5948	0.00	2,917	0.01
DNA	2760	0.04	47584	0.02	7,741	0.02
Unclassified	15716	0.24	996767	0.42	442,754	1.23
Small RNA	48,360	0.74	1,015,588	0.43		
Satellites	1,231	0.02	0	0.00	434	0.00
Simple Repeats	224,370	3.46	5,260,947	2.22	118,141	0.33
Relative volume	-	4.68	-	3.85	-	1.71
Common with Octopus	35,966	0.55	-	-	-	-
Squid only	157,034	2.42	-	-	-	-
Common with human repbase	224,370	3.46	-	-	-	-

Table S5 Repeatmasker output and comparison with *Octopus bimaculoides* and *Lottia gigantea*

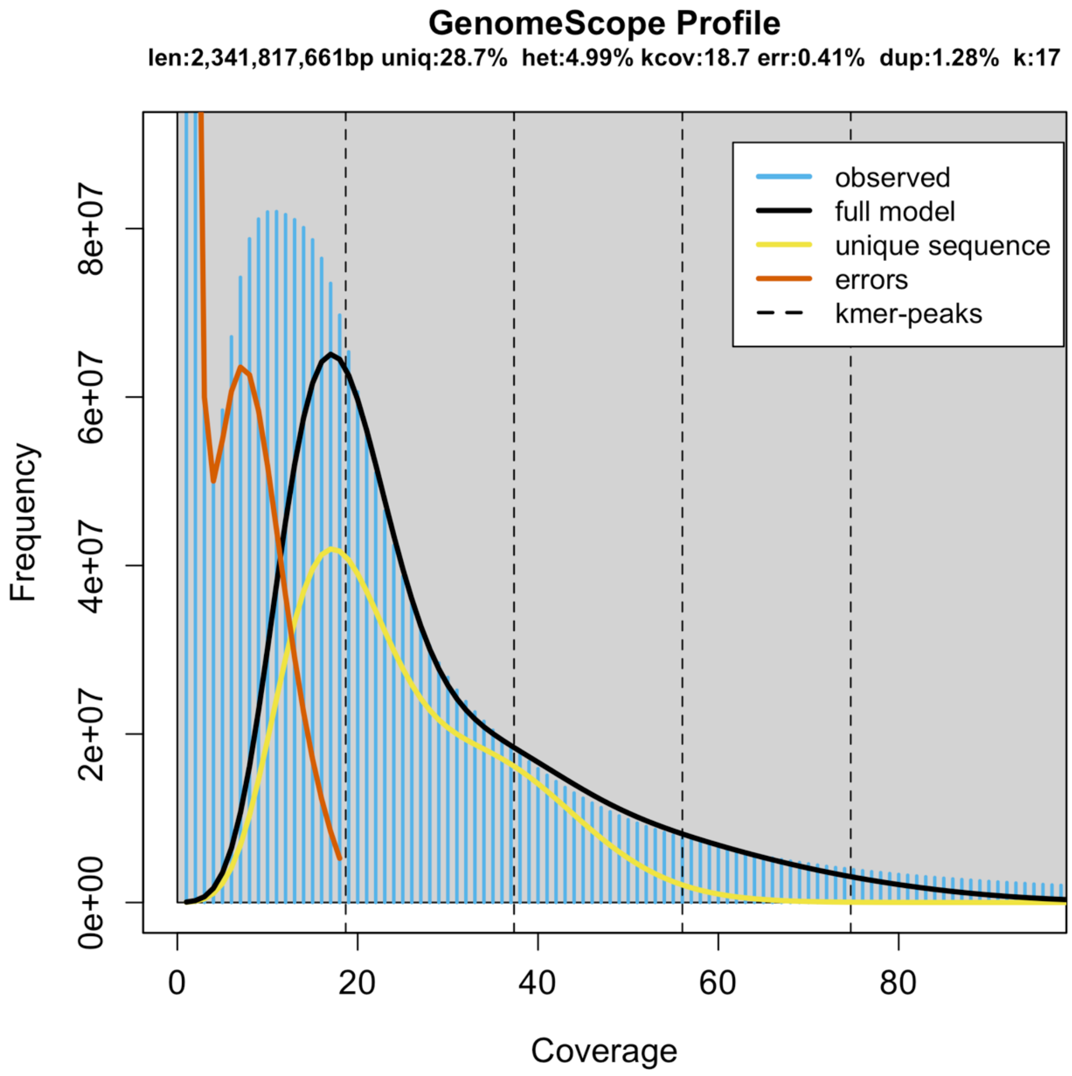

Figure S1 GenomeScope estimation profiles of k-mer distribution.

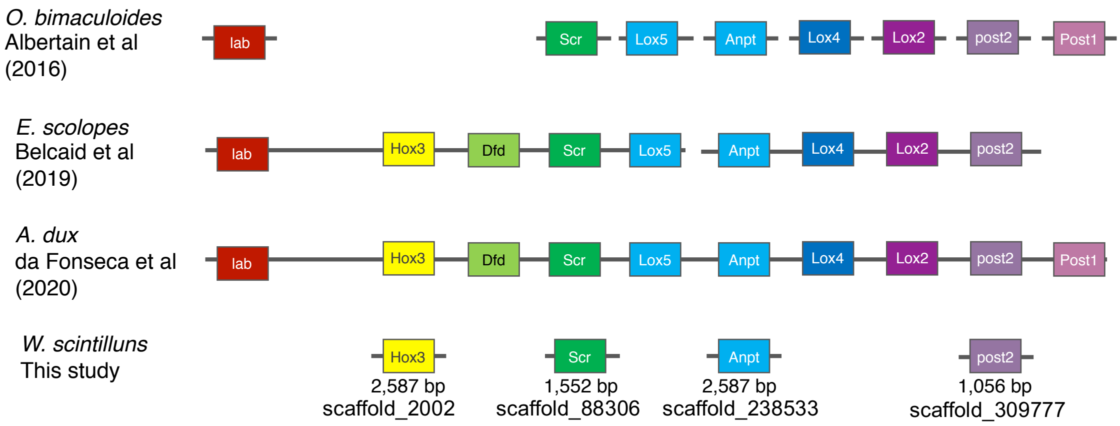

Figure S2 Schematic diagram of the HOX cluster gene created with reference to da Fonseca (2020). A single cluster of 10 HOX genes, excluding pb/HOX2, is the basic form of the cephalopods. *Watasenia* has a shares commonality with squid in terms of the HOX3 found in *A. dux* and *E. scolopes* but not in the octopus.

#genes	576
mean	10.7
SD	7.9
median	12

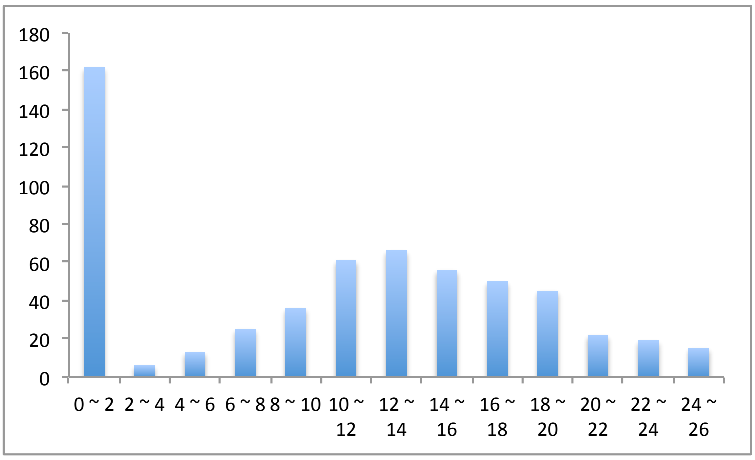

Figure S3 Unique gene coverage on BUSCO genes. The X-axis shows numbers of genes, Y-axis shows the coverage. Among 675 singleton BUSCO genes, values about 2σ away from the mean are considered as outliers and deleted. The peak was found at the 12-14 position, with a mean value of 10.7.

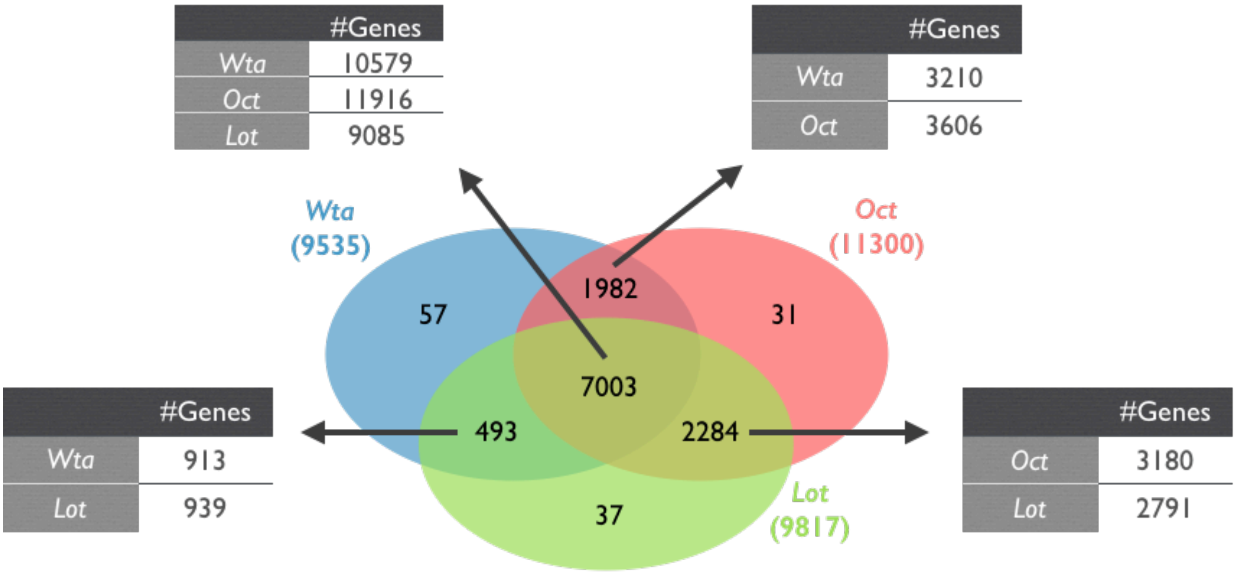

Figure S4 Orthologous analysis using three molluscan genomes, *Watasenia scintillans* (wta), *Octopus bimaculoides* (Oct) and *Lottia gigantea* (Lot). Venn diagram represents the sharing pattern of orthologous gene groups. The digit indicates the number of orthologous gene groups. Tables show the number of genes in the orthologous gene groups in each species. There should be an underestimate due to partialness of the *Watasenia* genome.

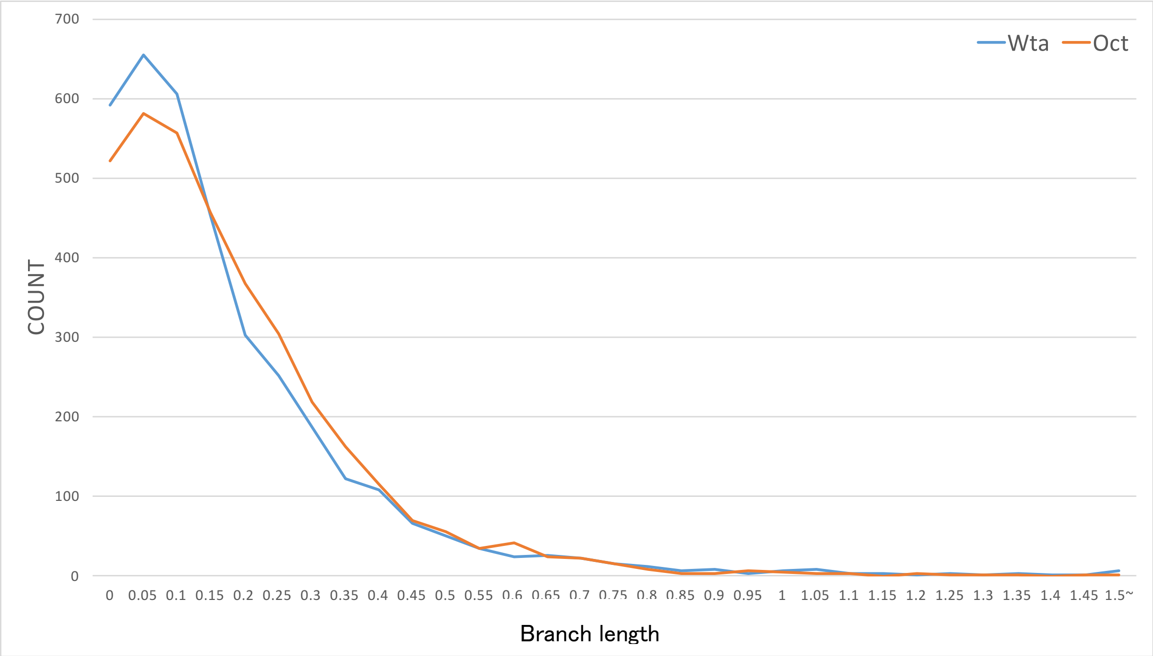

Figure S5 Sequence diversity from the common ancestor, *Lottia gigantea*.

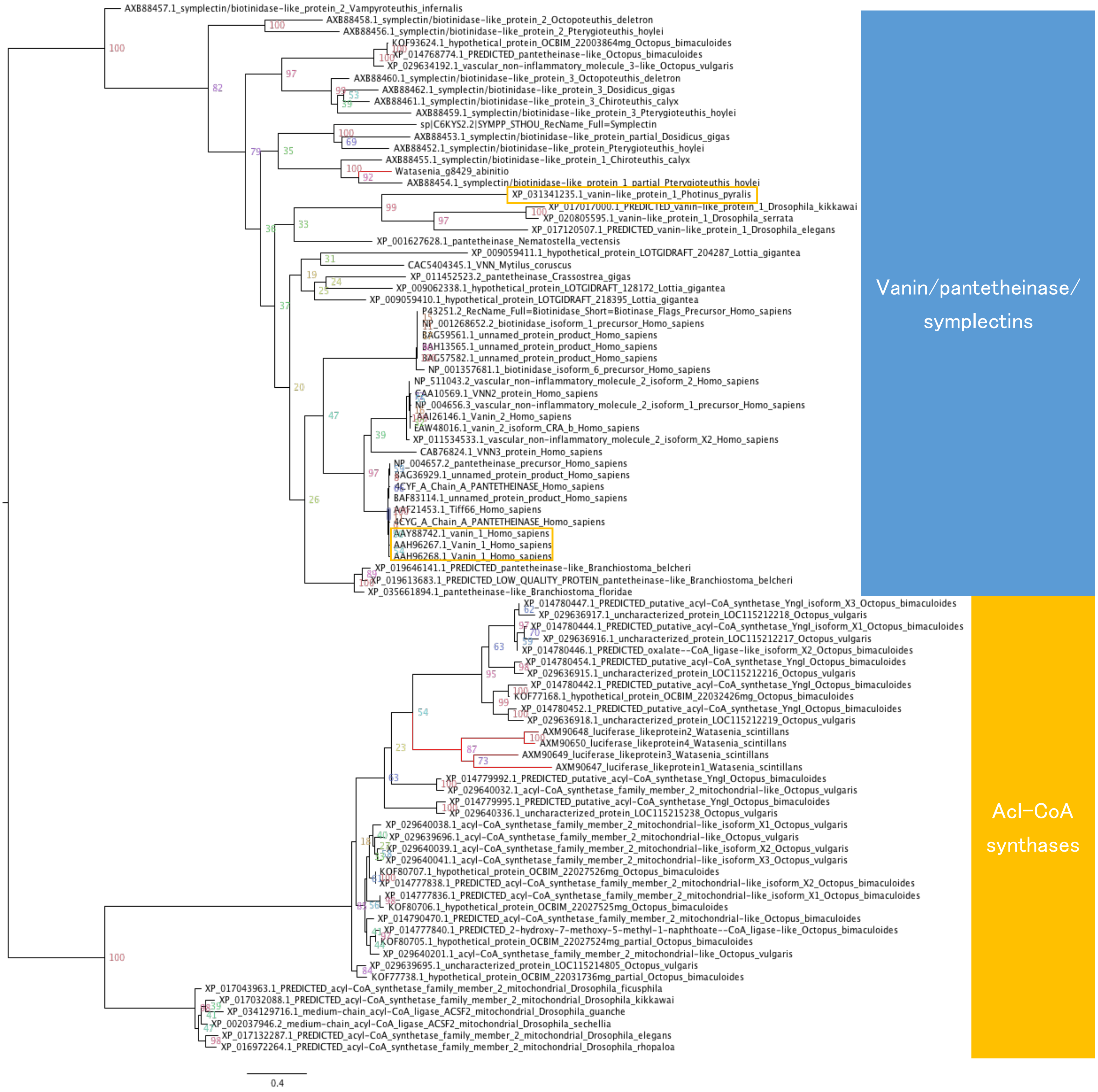

Figure S6 Phylogenetic analysis of animal luciferase homologs

A maximum likelihood tree was reconstructed from a multiple sequence alignment of lusiferases (see Materials and Methods). Nodes of *Watasenia* luciferases are shown in

red. Branch length represents substitutions per site. Bootstrap values with 1,000 repetitions are indicated on the nodes.

In this phylogenetic tree, there are two major groups, vanin and acyl-CoA synthase, with high support for each node; the Vanin cluster includes all the major lineages of cnidarians, spiralians, ecdysozoans, and deuterostomes, indicating that the gene is ubiquitous in the animal kingdom. *Photinus pyralis* (firefly) luciferase and human vanin-1, both of which are known to have 3D structures, belong to this cluster (surrounded by orange rectangles). The *Watasenia* symplectin-like (Watasenia_g8429_abinitio) forms a strong monophyletic group with symplectins of two open-eyed squid species (*Pterygioteuthis* and *Chiroteuthis*) and is included in the vanin cluster. The symplectins possessed by *Pterygioteuthis* do not form a monophyletic group within a species, unlike the enope squid luciferase described below, suggesting a large diversity of symplectins within a single species. This is also true for human vanins.

On the other hand, the acyl-CoA synthase is composed solely of the genomes of two octopus species and genes found in the enope squid and *Drosophila*. Since octopuses are not known to be luminescent and lack the luciferin coelenterazine, it is assumed that what is found in the octopus genome is the original enzyme that functions as an acyl-CoA synthase. The four species of firefly squid luciferase are closely related to each other, suggesting that they evolved from the acyl-CoA synthase in cephalopods and duplicated in the firefly squid. It is noteworthy that the genomic data did not have any hits with the bacterial luminescent squid, *Euprymna*.

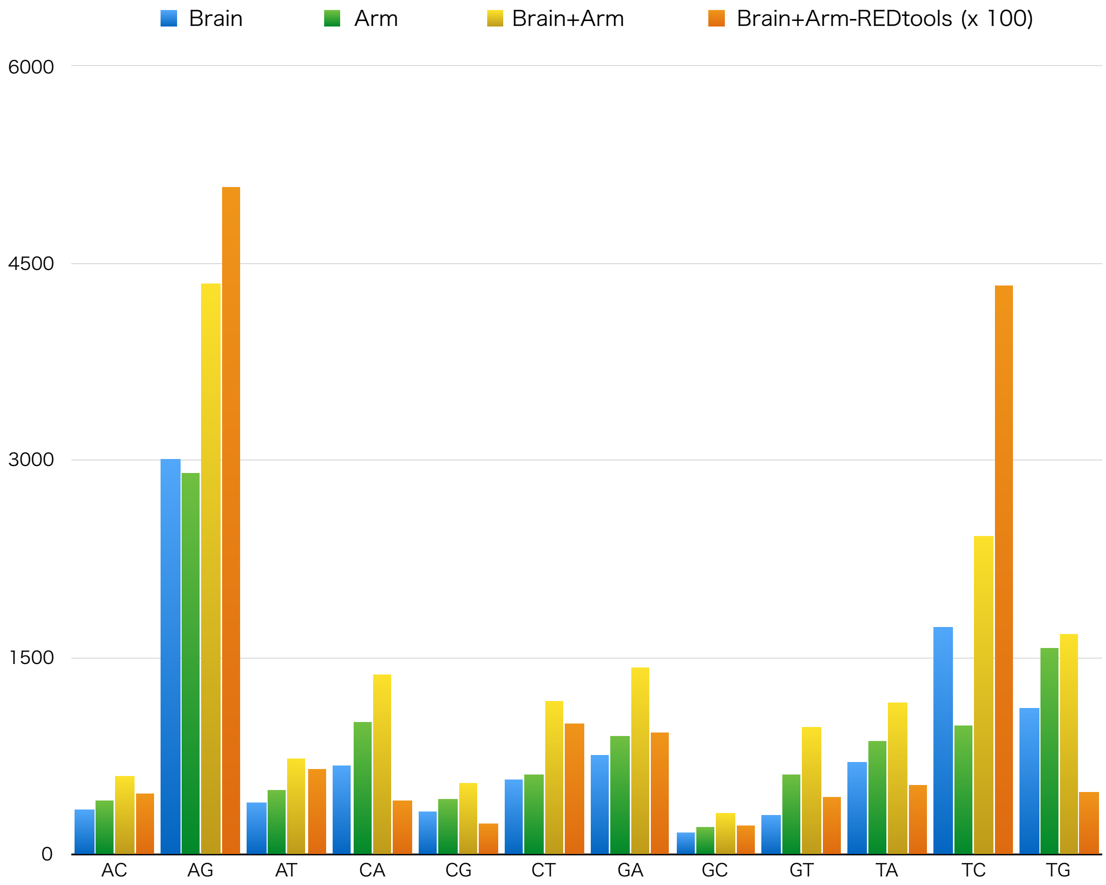

Figure S7 Comparison of RNA editing sites of Watasenia. **A.** A histogram showing distribution of edited residues. Adenine to inosine residues (A–I editing) in mRNAs carried out the most frequently. **B.** A Venn diagram of shared RNA editing sites between the brain and arm. RNA editing sites are not shared between the tissues.

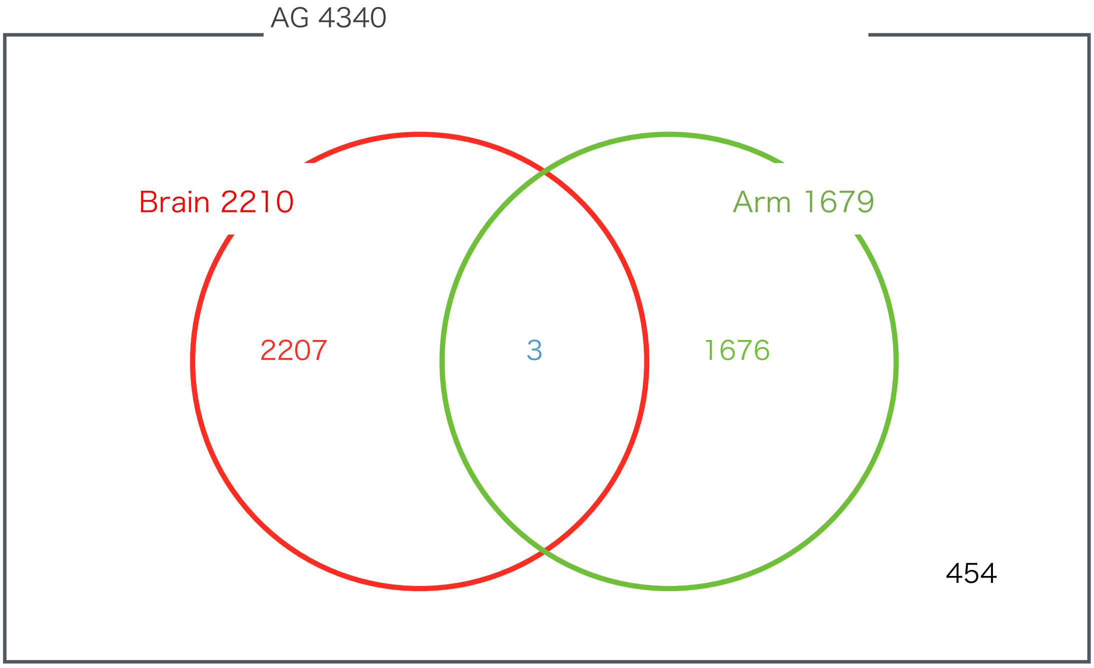

Figure S8 Comparison of RNA editing sites of Watasenia. **A.** A histogram showing distribution of edited residues from A to G (AG), which was came from Adenine to inosine residues (A–I) editing in mRNAs, carried out the most frequently. **B.** A Venn diagram of shared RNA editing sites between the brain and arm. RNA editing sites are not shared between the tissues.
